# Drug‐Based Lifespan Extension in Mice Strongly Affects Lipids Across Six Organs

**DOI:** 10.1111/acel.14465

**Published:** 2025-03-24

**Authors:** Sara Greenfield, Nathaniel C. Stevens, Lauren Bishop, Zachary Rabow, Daniela C. Soto, Abdali Omar Abdullah, Richard A. Miller, Oliver Fiehn

**Affiliations:** ^1^ West Coast Metabolomics Center University of California Davis California USA; ^2^ Department of Biochemistry & Molecular Medicine, MIND Institute University of California Davis California USA; ^3^ Department of Pathology and Geriatrics Center University of Michigan Ann Arbor Michigan USA

**Keywords:** aging, drugs, lipids, longevity regulation, mass spectrometry

## Abstract

Caloric restriction is associated with slow aging in model organisms. Additionally, some drugs have also been shown to slow aging in rodents. To better understand metabolic mechanisms that are involved in increased lifespan, we analyzed metabolomic differences in six organs of 12‐month‐old mice using five interventions leading to extended longevity, specifically caloric restriction, 17‐α estradiol, and caloric restriction mimetics rapamycin, canagliflozin, and acarbose. These interventions generally have a stronger effect in males than in females. Using Jonckheere's trend test to associate increased average lifespans with metabolic changes for each sex, we found sexual dimorphism in metabolism of plasma, liver, gastrocnemius muscle, kidney, and inguinal fat. Plasma showed the strongest trend of differentially expressed compounds, highlighting potential benefits of plasma in tracking healthy aging. Using chemical set enrichment analysis, we found that the majority of these affected compounds were lipids, particularly in male tissues, in addition to significant differences in trends for amino acids, which were particularly apparent in the kidney. We also found strong metabolomic effects in adipose tissues. Inguinal fat exhibited surprising increases in neutral lipids with polyunsaturated side chains in male mice. In female mice, gonadal fat showed trends proportional to lifespan extension effect across multiple lipid classes, particularly phospholipids. Interestingly, for most tissues, we found similar changes induced by lifespan‐extending interventions to metabolomic differences between untreated 12‐month‐old mice and 4‐month‐old mice. This finding implies that lifespan‐extending treatments tend to reverse metabolic phenotypes to a biologically younger stage.

## Graphical abstract

Using six interventions that have previously shown to differentially increase lifespan in male and female mice, we here show significant metabolic changes across organs, sex and metabolic pathways. Specifically, lipid responses were prominent across organs, but also in a sexually dimorphic manner.

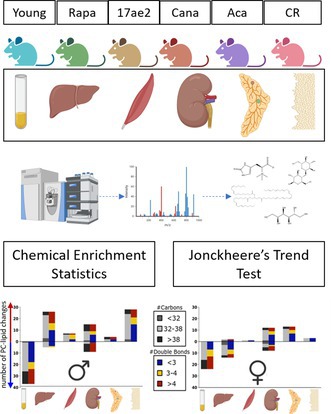

## 1 Introduction

Old age in humans is associated with many diseases, such as cardiovascular disease (North and Sinclair 2012), Alzheimer's disease (Guerreiro and Bras 2015), macular degeneration (Lim et al. 2012), infection (Kline and Bowdish 2016), and type 2 diabetes (Fletcher, Gulanick, and Lamendola 2002). However, some humans live extended lifespans without experiencing major diseases (Franceschi and Bonafè 2003). Understanding the mechanisms of aging rates and healthy aging might thus improve overall health. We here used mouse models to test the effects of interventions that extend lifespans in mice (Harrison et al. 2009, 2014, 2021; Miller et al. 2014, 2011, 2020; Strong et al. 2016; Weindruch 1996), specifically, caloric restriction (CR), drugs that affect similar metabolic pathways as caloric restriction like Rapamycin (Rapa), Acarbose (Aca) and Canagliflozin (Cana), in addition to 17‐α estradiol (17aE2) for which exact mechanisms of action are unclear (Harrison et al. 2014; Garratt et al. 2018, 2019). Rapa, Aca, and 17aE2 have also been shown to act on the MAPK pathway (Wink, Miller, and Garcia 2022). CR is the best studied way to slow aging and improve longevity in mice (McCay, Crowell, and Maynard 1989; Weindruch 1996). It has been shown to affect multiple metabolic pathways including the mechanistic target of rapamycin (mTOR), sirtuins (Wolf 2006), peroxisome proliferator activated receptor G coactivator‐1 (PGC‐1 alpha) (Anderson and Prolla 2009; Aghaei, Nilforoushzadeh, and Aghaei 2016), AMP‐activated protein kinase (AMPK) (Stancu 2015; Ge et al. 2022), and insulin/insulin growth factor‐1 (IGF1) (Chen et al. 2020; Testa et al. 2014; Karagöz and Gülçin Sağdıçoğlu Celep 2023). These pathways are involved in energy metabolism of fats and carbohydrates, and alteration of mitochondrial function.

Caloric restriction is difficult to maintain in humans and therefore has very limited clinical potential as an anti‐aging intervention. Thus, identification of drugs that can induce life extension is a next best avenue of research. Caloric restriction changes overall metabolism in many organs (De Guzman et al. 2013; Xie et al. 2022). We here investigated the metabolic effects of four drugs that are known to extend mouse lifespan in one or both sexes, using data from six mouse organs (plasma, liver, gastrocnemius muscle, kidney, and two adipose tissues: gonadal fat and inguinal fat). As the drugs induce varying sex‐dependent and sex‐independent differences in extending lifespan in mice, we performed a range of statistical tests, including trend analysis, for over 2000 metabolites analyzed by three different mass spectrometry methods. Using enrichment statistics, we then summarized the most important effects to 90 metabolite subclasses that were further categorized into 10 superclasses. While many metabolite subclasses demonstrated significant alterations under lifespan‐extending treatments, long‐chain polyunsaturated fatty acid‐containing complex lipids were most prominently associated with drug effects across different organs. Interestingly, effects in fat tissues were just as prominent as differences in blood and liver, while only modest impacts on muscle tissues were observed.

## 2 Methods

### 2.1 Mouse Husbandry

Male and female UM‐HET3 (Miller 2016; Miller et al. 1999) mice were all born, raised, and treated at the University of Michigan, from breeders purchased from the Jackson Laboratory. They were housed from weaning at three males/cage or four females/cage. Food and water were provided ad lib (except for the CR group). Light duration was controlled on a 12:12 cycle. Food was prepared using Purina 5LG6 as the base formula (Musi and Hornsby 2021). Twenty‐five male mice and 23 female mice were kept as controls, while 11–14 mice per sex per treatment were exposed to lifespan‐extending interventions (see Table S1). Mice were exposed to caloric restriction as follows: At 4 months, food consumption was measured in the controls, and the CR group was reduced to 20% calorie restriction afterward. At 4.5 months of age, the food consumption of the controls was re‐measured, maintaining the CR group at 20% calorie restriction. At 5 months age, food consumption was tested again, switching the CR treatment group to a 40% calorie restriction. The food consumption of the control mice was tested again every month from 6 to 11 months, and the CR group was kept at 40% calorie restriction. The CR group was fed once daily, including Saturdays and Sundays, between 8 AM and 9 AM. Drug treatments started at 4 months of age using UM‐HET3 mice and were administered with the food at 14.4 ppm (mg/kg food) for 17‐α estradiol (Steraloids, Newport, Rhode Island), 1000 ppm (mg/kg food) for acarbose (Spectrum Chemical Mfg. Corp., New Brunswick, New Jersey), 14 ppm (mg/kg food) for rapamycin (Emtora, San Antonio, Texas), and 180 ppm (mg/kg food) for canagliflozin (University of Michigan hospital pharmacy). Drug dosages were chosen based on previous work showing they increase mouse lifespan in UM‐HET3 mice (Harrison et al. 2009, 2021, 2019; Miller et al. 2011, 2014, 2020; Strong et al. 2016). Except for the CR group, mice had continuous access to food. Food intake of drug treated mice were not measured in this study. At 12 months, mice were euthanized through CO_2_ asphyxiation between 8 AM and 11 AM. Blood was then collected by closed‐chest cardiac puncture, and dissection and harvesting of other tissues followed. Samples were flash frozen and kept at −80°C until further processing. The young controls were sacrificed at 4 months. Other variables were not measured, but details are given on lifespan extensions before (Harrison et al. 2021, 2014; Miller et al. 2011, 2020). All procedures were reviewed and approved by the University of Michigan Institutional Animal Care and Use Committee.

### 2.2 Metabolomics Data Acquisitions

One milligram dry weight (DW) kidney, 2 mg DW liver, 3 mg DW gastrocnemius muscle, and 5 mg DW gonadal and inguinal fat were lyophilized, ground, and using 1 mL of a −20°C cold ternary solvent method of methanol, water and MTBE for liquid extraction (Matyash et al. 2008). In total, 20 μL of plasma was extracted in the same manner. Phase aliquots were used for three mass spectrometry assays, primary metabolites by GC‐TOF MS, biogenic amines by HILIC‐accurate mass MS/MS and lipidomics by RPLC‐accurate mass MS/MS (Appendix S1). Data processing used MS‐DIAL v. 4.48 (Tsugawa et al. 2015). Compound identification was performed by accurate mass and MS/MS spectral matching using MassBank of North America and NIST20 libraries (NIST, Gaithersberg, Maryland).

### 2.3 Statistics

Mann–Whitney *U* analysis was conducted using the python package scipy.stats (Virtanen et al. 2020), and the Benjamini–Hochberg *p*‐value correction was conducted using the python package statsmodels (Seabold and Perktold 2010). Chemical enrichment analysis was conducted using ChemRich (Barupal and Fiehn 2017). Jonckheere's trend test was conducted using the R package clinfun, using the function jonckheere.test (Seshan and Whiting 2023).

## 3 Results

### 3.1 Lifespan‐Extending Interventions Change Metabolite Levels in All Organs

**FIGURE 1:**
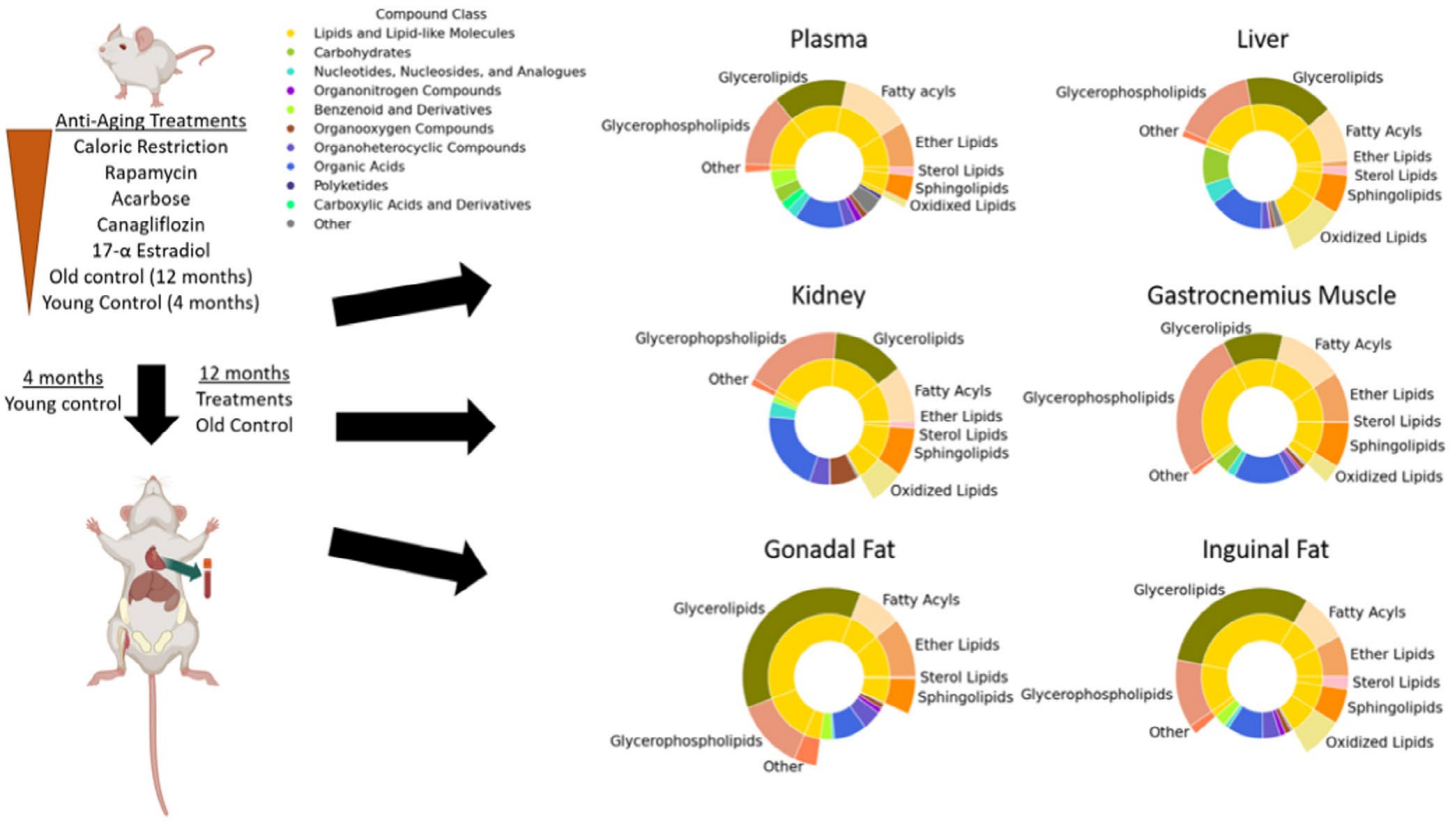
Study design and classes of identified compounds per organ. Mice were treated with caloric restriction, rapamycin, acarbose, canagliflozin, or 17aE2 interventions from 4 to 12 months of age, with an additional comparison with organs of 4‐month‐old controls. Metabolomic assays focused on lipidomics by charged surface hybrid liquid chromatography‐accurate mass spectrometry (CSH), primary metabolism by gas chromatography‐time of flight mass spectrometry (GC‐TOF), and biogenic amines by hydrophilic interaction liquid chromatography‐accurate mass spectrometry (HILIC). Circles represent the proportions of structural compound classes of the identified compounds across all controls and interventions for each tissue, as classified using ClassyFire (Djoumbou Feunang et al. 2016). The inner circles show main structural classes of all compounds, and the outer circles show lipid subclasses.

To understand the effects of five lifespan‐extending treatments in 12‐month‐old mice, we used four drug interventions and caloric restriction (Figure 1), with an additional comparison with untreated 4‐month‐old mice (Young Control). The average extent of increased lifespan differed by the type of intervention, the majority being reportedly stronger in males than in female mice (Harrison et al. 2021, 2009, 2014; Strong et al. 2016; Miller et al. 2014, 2011, 2020). Sorted by the percent of increased lifespan, interventions yielded different lifespan extensions in female and male UM‐Het3 mice (Table 1). Additional data of the effects of these lifespan extension effects have been published. Here, we only used average lifespan extension data from studies that most closely represented our study design (Table 1). Across all tissues and treatments, the three metabolomic assays yielded a set of 2551 chemically annotated metabolites (Appendix S2). Compound classifications showed expected differences between organs, such as a higher proportion of carbohydrates in the liver and a larger ratio of organic acids in the kidney, with the lowest amounts of organoheterocyclic compounds in the liver (Figure 1). Adipose tissues had the greatest proportion of reported lipids, specifically neutral lipids except sterol lipids, while liver and kidney exhibited the lowest ratio of ether lipids and muscle showed the highest proportion of phospholipids (Figure 1). Unidentified compounds were not statistically evaluated in this report.

**TABLE 1:** Average lifespan extensions by drug or caloric restriction interventions compared to control mice in UM‐Het3 mice, in order of increasing lifespan‐extending effect (Harrison et al. 2009, 2014, 2021; Miller et al. 2014, 2011, 2020; Strong et al. 2016; Weindruch 1996).

Female	Control (0%)	17aE2 (1%)	Cana (1%)	Aca (5%)	Rapa (21%)	CR (40%)
Male	Control (0%)	Rapa (13%)	Cana (14%)	17aE2 (19%)	Aca (22%)	CR (33%)

**FIGURE 2:**
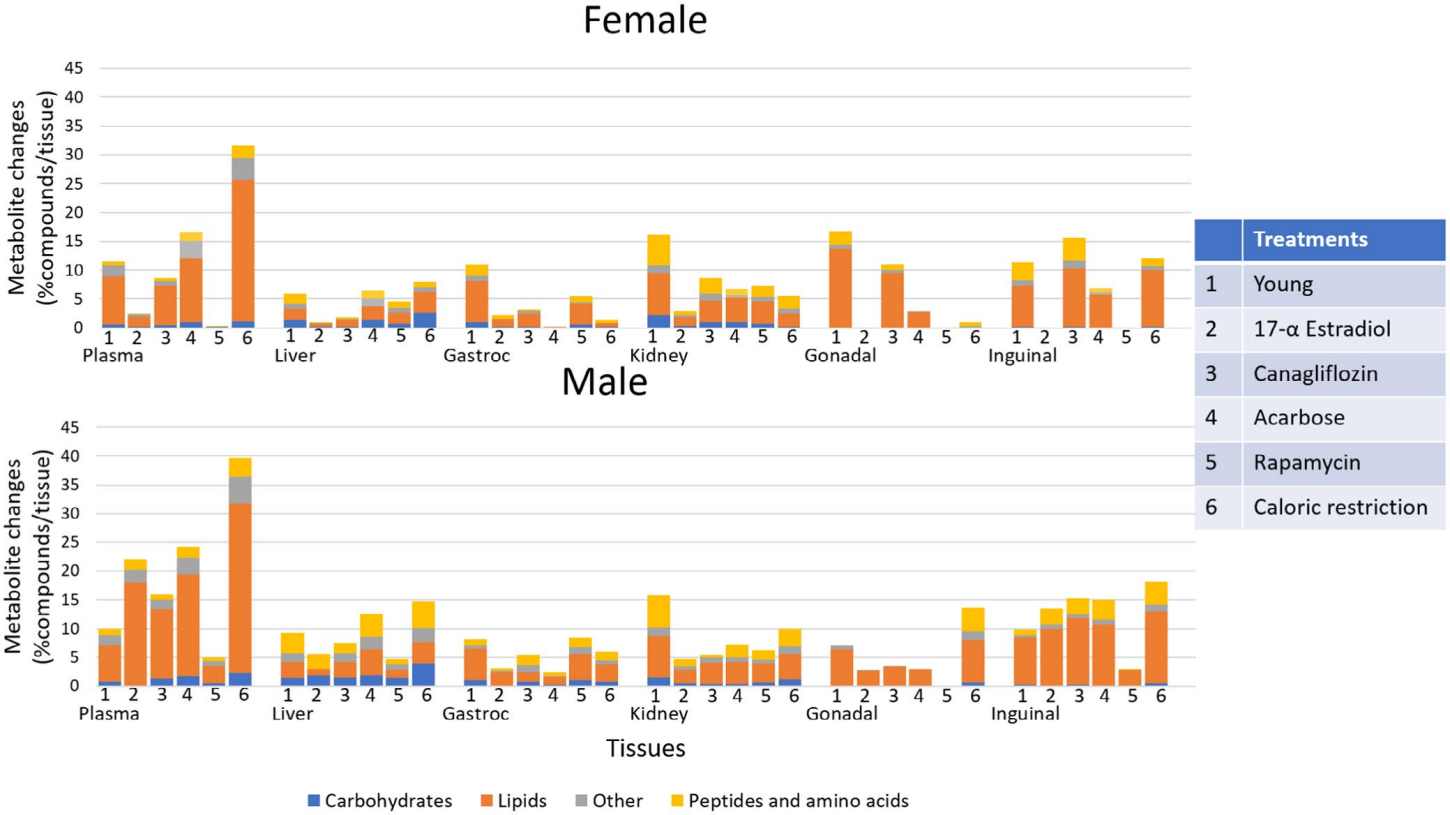
Relative impact of interventions on mouse organ metabolism by univariate analyses. Per organ, the proportion of significantly changed metabolites (FDR *q* < 0.1) is given in comparison with 12‐month mice, summarized into chemical superclasses. Metabolite levels measured from organs of treated mice and 4‐month‐old controls (Young) were compared to 12‐month‐old control mice, using Benjamini–Hochberg corrected Mann–Whitney *U* tests at *q* < 0.1. Treatments are ordered by increasing life extension effects for both female and male mice as listed in Table 1, starting with 4‐month‐old mice as a reference for biologically younger mice. Data are available in Appendix S2.

We then explored the generic effects of interventions on 12‐month‐old mice by comparing metabolite levels measured from organs of treated mice to age‐matched control mice. Using Benjamini–Hochberg corrected Mann–Whitney *U q* < 0.1, we found the most compounds to be differently regulated by interventions in plasma from caloric restriction treated male mice (Figure 2). Interestingly, for all organs except plasma, the proportion of metabolomic changes by interventions were similar or smaller than the differences observed when directly comparing 12‐month‐old mice to 4‐month‐old mice (Figure 2). The superclasses in the 12‐ versus 4‐month age comparisons are similar to responses observed in response to longevity‐extending interventions, with lipids as the most affected class of compounds, followed by peptides and amino acids. Some interventions did not yield any significant changes in specific organs, for example, rapamycin for female adipose tissues and plasma (Figure 2). Our data suggest that the largest proportion of metabolic changes was found in lipids, especially in plasma, followed by peptides in kidney and liver (Figure 2). As expected, caloric restriction exerted the largest effect on metabolite levels for both sexes, particularly in plasma. Surprisingly, except for in gastrocnemius muscle, Rapamycin yielded few significant effects. Acarbose showed a large number of significantly changed compounds in fat, liver, kidney, and plasma (Figure 2). In females, 17aE2 does not lead to significant longevity and, correspondingly, it also did not show metabolic changes (Table 1, Figure 2). However, for males, 17aE2 treatments changed > 20% of all compounds in plasma and > 10% of all compounds in inguinal fat and liver, followed by other organs. This finding indicated that differences in lifespan extension effect caused by drug interventions may be observed by alterations in organismal metabolism.

**FIGURE 3:**
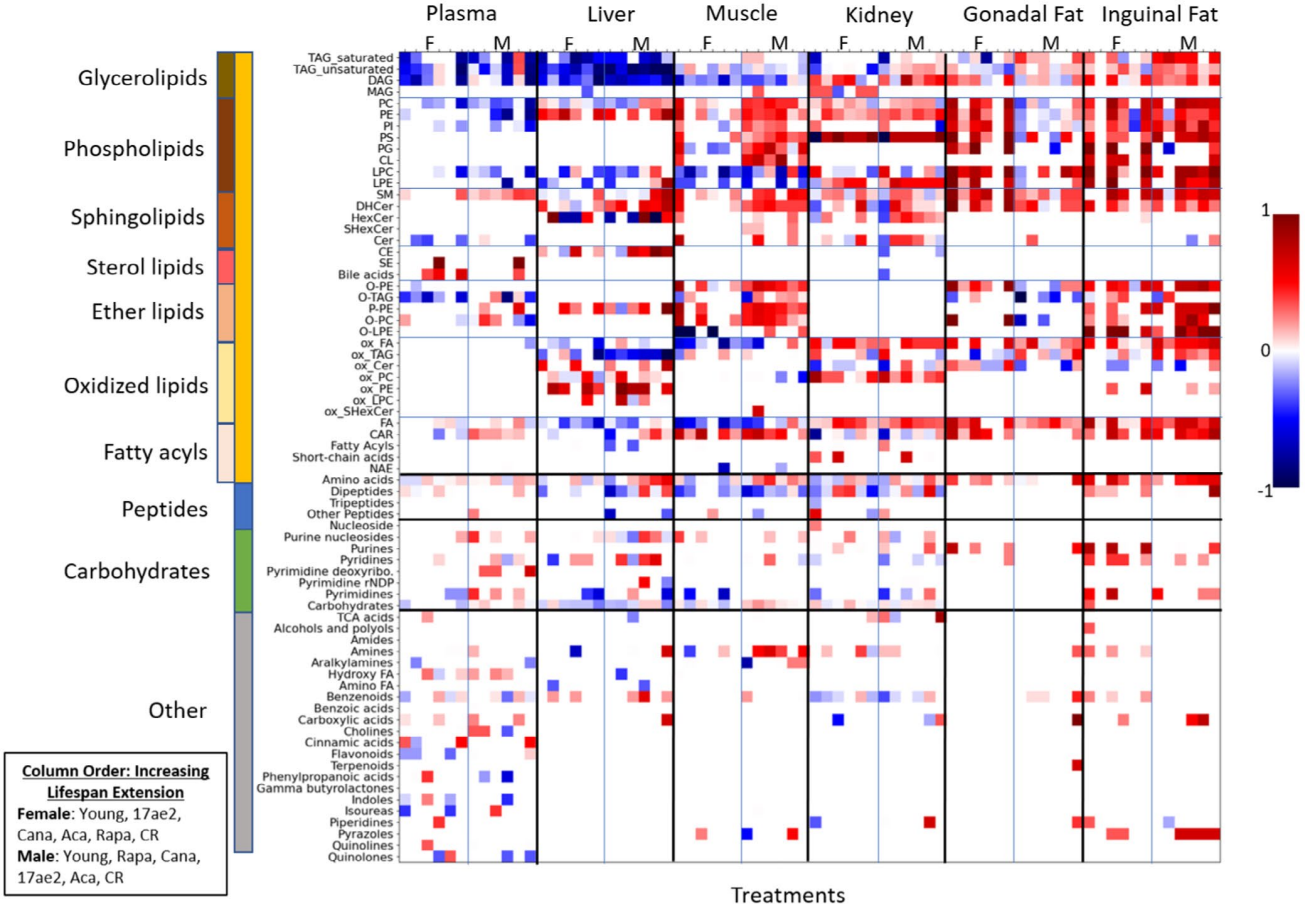
Effects of interventions on mouse organ metabolites by chemical set enrichment analyses. Raw *p*‐values from univariate statistical differences for interventions in 12‐month‐old mice were summarized into ChemRICH set statistics at Kolmogorov–Smirnov *p* < 0.05. To highlight classes with strong directional trends, we then subtracted the number of compounds significantly down regulated from the number significantly upregulated, and divided that number by the total number of compounds in that class for that tissue. Differential regulation is shown for primarily upregulated classes (red) versus primarily downregulated classes (blue). Color intensities indicate the percentage of differentially regulated compounds in the majority direction per class. Treatments are separated by sex and ordered by increasing life extension effect of treatment as listed in Table 1, starting with 4‐month‐old mice as a reference for biologically younger mice in each organ/sex column. Data are available in Appendix S3.

Next, we investigated these metabolomic changes by more detailed chemical classifications into ClassyFire subclasses and used Chemical Set Enrichment statistics (Barupal and Fiehn 2017) to summarize the results of univariate Mann–Whitney *U* statistics (Figure 3). Set enrichment statistics of structural subclasses yielded much better details on the differential regulation of metabolites across mouse organs than organization by superclass as in Figure 2. For most compound classes and across all tissues, the patterns noted in comparisons of 4‐ to 12‐month‐old control mice were similar to those seen in comparison between the longevity‐intervention groups compared to 12‐month controls. This is particularly apparent when comparing the metabolomic changes induced by caloric restriction (the far‐right panels in each organ/sex column) to the 4‐month control mice (the far‐left panels in each organ/sex column).

The effect of lipid metabolomic alterations in life‐extending interventions remained dominant in comparison with peptides, carbohydrates, or other metabolite subclasses. Additionally, these interventions appear to affect specific types of lipids preferentially. For example, neutral lipids, specifically di‐ and triacylglycerides, were downregulated for both male and female mice in plasma and liver, but were found mostly increased in adipose tissues. In contrast, cholesterol esters (CE) remained mostly unaffected by any intervention, except for the liver. In the liver, regulation was opposite to effects on triacylglycerides, suggesting that effects are unlikely to be explainable by external factors such as lipoprotein abundance (in blood) or method biases (for extraction efficiency). Interestingly, monoacylglycerols also did not show intervention effects, indicating that tri‐ and diacylglycerol lipases were differentially affected by treatments, but not all lipases were affected, including monoacylglycerol lipases.

Similarly, we found tissue‐specific effects on phospholipids. In plasma and liver, limited differential effects were found on phospholipids in response to life‐extending treatments. In comparison, much stronger upregulation of several phospholipid classes was found in male muscle tissue, female gonadal fat, and inguinal fat for both sexes. Correspondingly, for specific treatments, a trend of decreasing amounts of lysophospholipids was found in muscle and liver, with sporadic events for plasma. Indeed, we observed the same trend for free fatty acid content in the liver and muscle. In combination, these data show that phospholipid lipase activity was decreased by the drug and caloric restriction interventions in muscle and liver. Conversely, both lysophospholipids and free fatty acids showed increasing associations with lifespan extension effect in inguinal fat, with similarly increasing patterns for gonadal fat and kidneys. We also observed higher amounts of complex phospholipids for the adipose tissues and kidney.

Hence, it appears that overall amounts of lipids were increased in these tissues, ranging from complex lipids to lyso‐lipids and free fatty acids. While an overall activation of deposition and use of these lipids is a possibility, neutral lipids (fats and diacylglycerides) showed this trend mostly for male mice, but not for female mice. Instead, as phospholipids are primarily membrane lipids, the increased trends for phospholipids may point toward a generic increase in cell numbers rather than differential regulation of specific lipases or biosynthesis. This view is supported by very similar patterns observed for ether‐base phospholipids that are produced in peroxisomes. This finding indicates that the lifespan‐extending interventions did not specifically affect peroxisomes. Interestingly, we found opposing trends for increasing levels of acylcarnitines accompanied by decreasing free fatty acid, lysophospholipid, and diacylglyceride levels in the muscle, caused by exposure to lifespan‐extending drugs. Such effects could point to improving beta‐oxidation (as fatty acid levels fall) simultaneously with increased fatty acid export through acylcarnitines. Surprisingly, we found a striking increase in phosphatidylserine levels in the kidney for all treatments, in addition to male muscle tissue. Little is known about phosphatidylserine functions in the kidney, including its precise subcellular localization, transmembrane topology, and intracellular dynamics (Kay and Fairn 2019).

While oxidative damage has long been discussed as a contributing factor in aging (Bokov, Chaudhuri, and Richardson 2004; Liguori et al. 2018), our investigation did not find a unifying trend for differential regulation of oxidized lipids across organs and treatments. Instead, we found an interesting pattern of differences between organs. In the liver, oxidized triacylglycerides were found at decreasing levels for most lifespan‐extending treatments, while the opposite trend was found for inguinal and gonadal fat. Notably, in the liver, we found a large increase in oxidized phosphatidyl‐ethanolamines, but we did not see this association for phosphatidyl cholines. Finally, we saw reduced oxidized free fatty acids in liver and gastrocnemius muscle, but increases in kidney and adipose tissues. These different trends across organs make it unlikely that sample treatments during analytical chemistry accidentally produced such oxidation damage. We have limited knowledge of whether partially oxidized lipids exert specific cellular functions, although such possibilities have been discussed, particularly in situations of caloric deficit (Gerhart‐Hines et al. 2007; Nakamura, Yudell, and Loor 2014). At a minimum, our data suggest that an alteration in lipid oxidation is unlikely to be a main driver for increased longevity across all organs.

For carbohydrates, only very moderate differences were found, with a small number of compounds found decreased in the liver (Jové et al. 2014). In comparison, the moderate changes in the levels of soluble sugars support the idea that changes in lipid metabolism are a main metabolic consequence of lifespan‐extending interventions in mice. Finally, we addressed the hypothesis that reduced muscle loss and reduced protein turnover could be a major driver for increased lifespan. Indeed, we found a general decrease in dipeptide levels in liver, muscle, and kidney associated lifespan‐extending interventions compared to age‐matched controls. Interestingly, free amino acids showed increased trends, similar to previously published reports (Gibbs et al. 2018), possibly due to an increased import into cells as energy supply.

### 3.2 Using Jonckheere's Trend Test to Associate Metabolite Classes With Increased Lifespan

Drugs and caloric restriction interventions may have metabolic effects on tissues that are not directly associated with the extension of average lifespan in mice. For example, 17aE2 and Cana interventions have been reported to cause no discernible increased lifespan in female mice, but we show they affect levels of many metabolites (Appendix S2). To better understand which metabolic differences might be functionality related to increased lifespan, we used the Jonckheere's trend test. This test, also called the Jonckheere‐Terpstra test, can be used to determine the significance of a trend in data, that is, to investigate whether an increase in one variable (e.g., lifespan extension effect) results in an increase or decrease in another variable, in this case the intensity of a metabolite showing a statistically significant difference in mice proportional to life extension effects of treatments. Here, we applied the Jonckheere's trend test to each compound in each tissue for the age‐matched controls and their corresponding interventions in the order of increasing life extension effect (Table 1). We then used the Benjamini–Hochberg false discovery rate (FDR) method for multiple testing correction.

**FIGURE 4:**
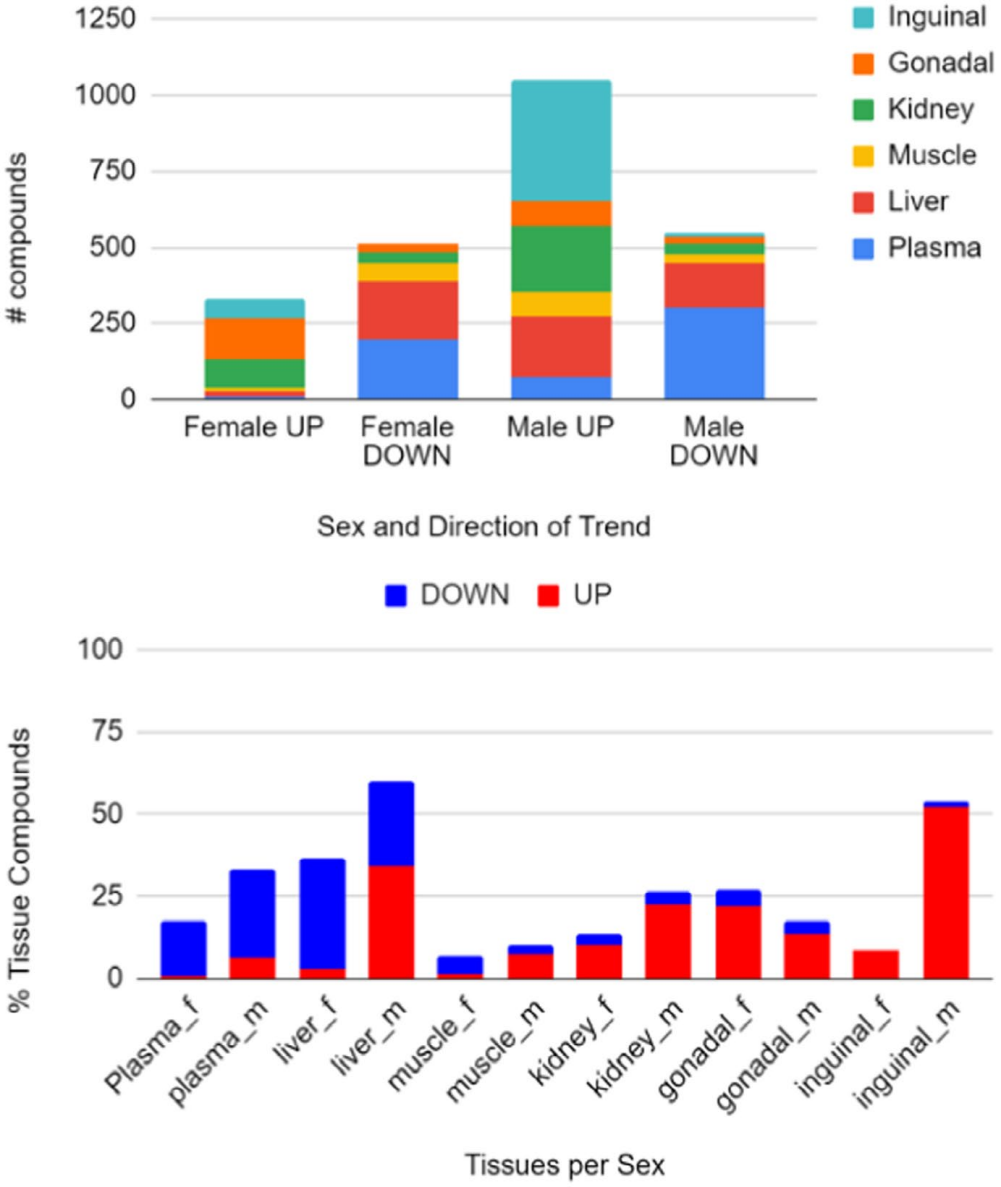
Number of metabolite changes across lifespan‐extending interventions. Median percent extensions of interventions were used for Jonckheere's trend test statistics at FDR *q* < 0.1. (Top) Total compounds showing effect across all tissues per sex (UP: Metabolite levels increasing proportional to life extension effect. DOWN: Metabolite levels decreasing proportional to life extension effect). (Bottom) Grouped by tissue and sex (red: Metabolite levels increasing with lifespan effect; blue: Metabolite levels decreasing with lifespan effect).

Overall, 1187 compounds in male and 729 in female of the total of 2551 annotated metabolites showed a significant trend at FDR *q* < 0.1 in at least one organ and sex (Appendix S2). This test highlighted sexual dimorphism. More metabolites showed significant trends with increasing lifespan extension in males than in females (Figure 4, top). Among those in male mice, more compounds showed positive associations with longer lifespan (“up”) than negative associations (“down,” Figure 4, top).

In most tissues, the number of compounds that showed increased levels with increasing lifespan was higher than the number of compounds that showed decreasing trends between metabolite levels and the degree of lifespan extension (Figure 4, bottom). Notably, this was not the case for plasma, where most compounds showed decreasing trends, similar to female liver and muscle tissues. The number of positively lifespan‐associated compounds in inguinal fat was far higher in male than in female mice. Indeed, for all organs except gonadal fat, males showed more compounds significantly associated with increases in lifespan (Figure 4, bottom). The highest number of significant compounds was found in male liver tissues (with both positive and negative associations), followed by male inguinal fat. As three of the five lifespan‐extending treatments have been shown to have a greater effect on male mice than female mice (Table 1), it is possible that this sexual dimorphism is a reflection of sex‐specific difference in drug action.

### 3.3 Neutral Lipids Are Most Strongly Associated With Lifespan‐Extensions in Mice

**FIGURE 5:**
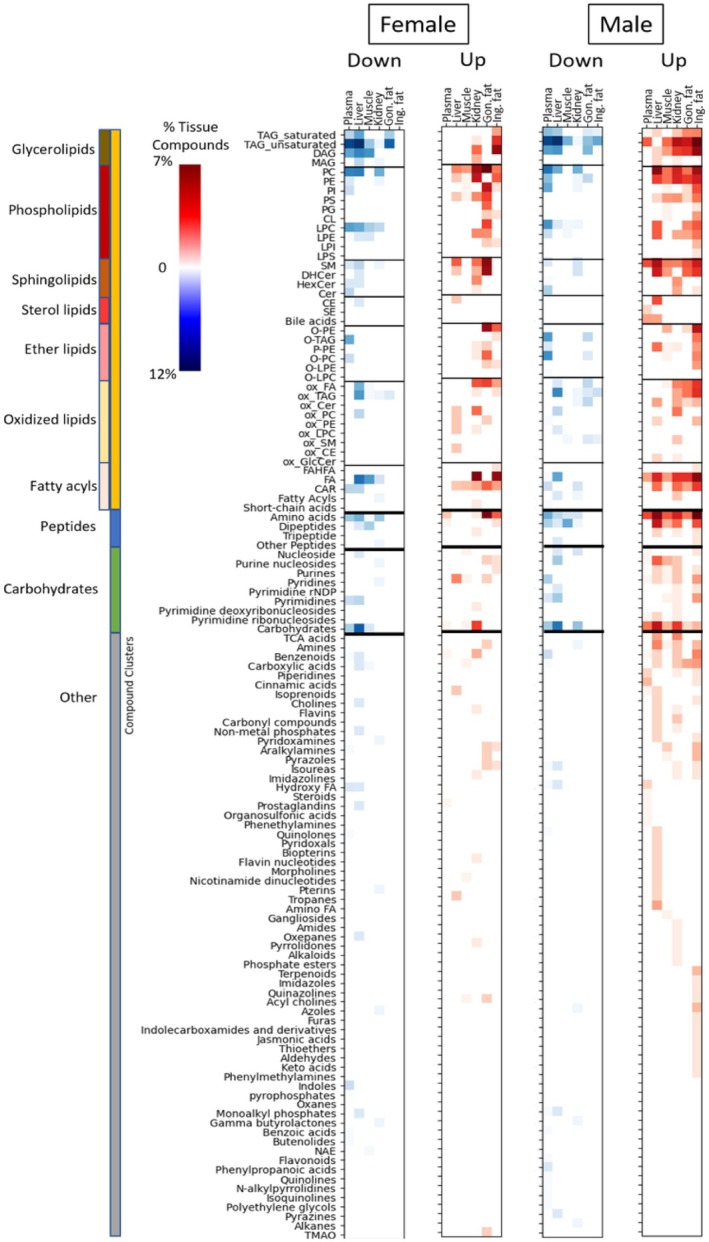
Chemical classes of metabolites changing across ordered lifespan‐extending interventions. Average lifespan extensions of interventions were used for Jonckheere's trend test statistics at FDR *q* < 0.1. Compounds were grouped into chemical categories by ClassyFire and normalized to the total number of compounds per tissue. Increasing trends (“up”) labeled in red, decreasing trends (“down”) given in blue. Color scales indicate the relative proportion of significant compounds per class.

Next, we wondered which compound classes were most prominently associated with increased lifespan in mice, and in which tissues. Here, we grouped the compounds into chemical classes using ClassyFire software (Djoumbou Feunang et al. 2016) and normalized their relative proportions to the total number of compounds in each tissue. Most importantly, we found an abundance of lipids to be associated with metabolic differences caused by lifespan‐extending interventions (Figure 5).

**FIGURE 6:**
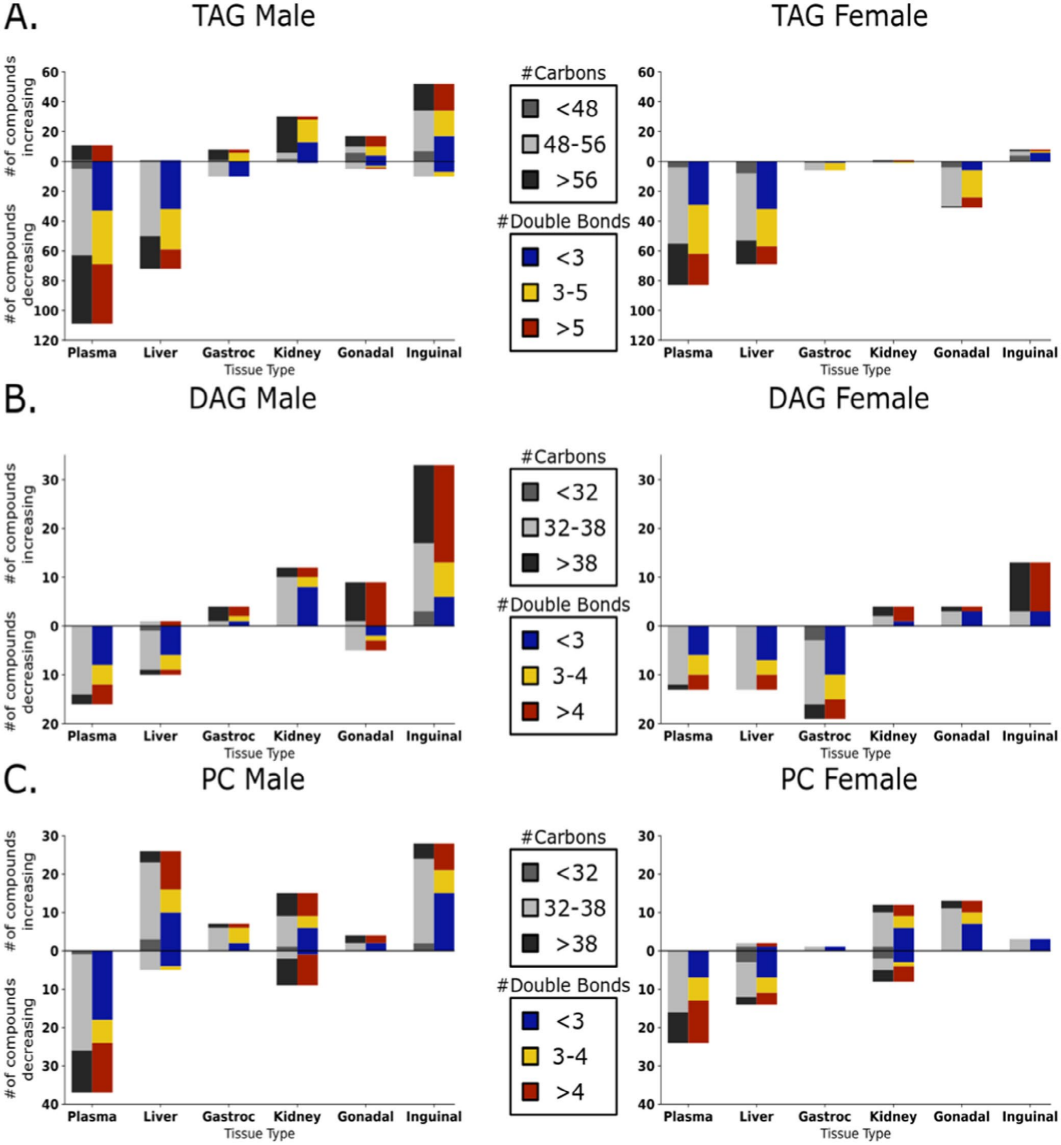
Subclassification of changes of triacylglycerides, diacylglycerides, and phosphatidylcholines across lifespan‐extending interventions. Average lifespan extensions of interventions (Table 1) were used for Jonckheere's trend test statistics at FDR *q* < 0.1. (A) Triacylglycerides (TAG) were differentiated between very‐long‐chain containing lipids (red: With acyl chains longer than 20 carbons, total > 57 carbons), classic long‐chain lipids (gray: With acyl chains 16–18 carbons, total 48–56 carbons), and short‐chain lipids (blue: With acyl chains < 16 carbons, total < 48 carbons); TAG classifications by the number of double bonds. Red: Enriched in polyunsaturated fatty acids. Yellow: Total of 3–5 double bonds per TAG. Blue: Enriched in saturated fatty acids with a total of 0–2 double bonds per triacylglyceride. (B, C) Subclassification of changes of diacylglycerides (DAG) and phosphatidylcholines (PC) across lifespan‐extending interventions. DAGs and PCs were differentiated between very‐long‐chain containing lipids (red: With acyl chains longer than 20 carbons, total > 38 carbons), classic long‐chain lipids (gray: With acyl chains 16–18 carbons, total > 32 carbons), and short‐chain lipids (blue: With acyl chains < 16 carbons, total < 32 carbons). DAG and PC classifications by the number of double bonds. Red: Enriched in polyunsaturated fatty acids. Yellow: Total of 3–4 double bonds per lipid. Blue: Enriched in saturated fatty acids with a total of 0–2 double bonds per lipid.

Specifically, many neutral glycerolipids, both triacylglycerides and diacylglycerides, showed a decreasing trend across tissues, most strongly in plasma and liver (Figure 5). An opposite, increasing trend, was found for neutral lipids in kidneys, inguinal fat and gonadal fat tissues (Figure 5). These observations support the notion that lower levels of circulating glycerolipids are indicative of better health, including lower fatty liver levels, with a better accumulation of storage lipids in adipose tissues. To better understand if specific subclasses of neutral lipids were associated with extending lifespans, we further broke down the lipid clusters by carbon number and fatty acyl desaturation levels (Figure 6). In male mice, we found in liver and plasma that of the triacylglycerides that showed negative association with lifespan, more than half were polyunsaturated fatty acyl groups (PUFA, defined as having at least three double bonds), mostly with long‐chain or very‐long‐chain carbon lengths (Figure 6A). In male kidneys, almost all positively associated triacylglycerides consisted of very‐long‐chain acyl groups (total carbons > 57). Conversely, in both adipose tissues, these triacylglycerides were found to be positively associated with longevity. In female mice, similar effects were found for plasma and liver, but no effects were seen in the kidneys. In contrast to male tissues, however, female gonadal fat showed decreasing trends of PUFA‐containing triacylglycerides (Figure 6A).

Adipose tissues use lipases to release free fatty acids from complex lipids that can be used in mitochondrial oxidation via albumin transport. Correspondingly, we found positive associations of free fatty acid levels with percent median lifespan extension in males across all organs (Figure 5), and in inguinal fat and kidney in female mice. The remaining diacylglycerides or lysophospholipids have been ascribed a range of physiological functions (Tan et al. 2020; Eichmann and Lass 2015). We found many PUFA‐containing diacylglycerides to be specifically upregulated in inguinal fat tissues for both male and female mice in proportion to degree of lifespan increase (Figure 6B). Conversely, mostly saturated and mono‐unsaturated diacylglycerides were negatively associated with lifespan extension in both plasma and liver for both sexes (Figure 6B). Thus, our data show a strong and sustained differential regulation of fat mobilization, storage, and transport marked by decreases of glycerolipids in liver and plasma, including decreased saturated and mono‐unsaturated diacylglycerides. In the liver, oxidized triacylglycerides (ox_TAG) and oxidized free fatty acids (ox_FA) showed lower levels proportional to degree of life extension in both sexes, but higher levels in kidneys and adipose tissues (Figure 5). Interestingly, these associations were completely absent in plasma.

### 3.4 Phosphatidylcholines Revealed Sexual Dimorphism in Response to Lifespan‐Extending Interventions in Mice

Phospholipids showed positive associations between metabolite levels and longevity increases across all tissues except plasma (Figure 5). This trend was particularly pronounced in male mice. Lysophosphatidylcholines diminished with higher lifespan effects in all organs except the adipose depots and male liver tissues, and female gonadal fat. Ether‐based phospholipids showed a pattern similar to other phospholipid classes, making it unlikely that peroxisomes (the organelle in which ether‐bonds are synthesized) play a specific role in these lifespan‐extending treatments. Phospholipids are also under constant regulation and exert specific functions in different organs. We found negative associations of phosphatidylcholines (PC), the largest class of phospholipids, with extent of lifespan extension in both sexes in plasma (Figure 6C). Here, we saw decreases for both saturated and polyunsaturated fatty acyls groups, mostly with classic long‐chain carbon lengths. These findings are consistent with observations of decreased levels of plasma lipoproteins and may help to explain the decreased levels of fat transport (albeit, specifically for long‐chain‐ and very‐long‐chain PUFA‐containing fats, see above). Other organs showed more complex regulation of PC lipid modulation under lifespan‐extending interventions (Figure 6C). Here, we found striking sexual dimorphism between female and male mice. For example, in liver tissue, many PC lipids were increased in males, but decreased in females exposed to lifespan‐extending interventions. Interestingly, most PC lipids that showed positive associations in male liver were PUFA‐containing long‐chain lipids. In contrast, females did see decreases in PC liver lipids under lifespan‐extending treatments, but these mostly excluded PUFA‐containing long‐chain lipids (Figure 6C). PUFA‐acyl chains change membrane fluidity and therefore alter cellular and organelle characteristics, which may contribute to effects of these interventions on longevity. A similar sexual dimorphism was found for adipose tissues. PC lipids showed positive associations with lifespan in male inguinal fat, but not in females. Conversely, in female perigonadal adipose tissue, PC lipids were upregulated under lifespan‐extending interventions, but not in males. Overall perigonadal adipose tissue had the greatest number of significantly positive associations in female mice (Figure 4, bottom). These lipids primarily consisted of phospholipids and di‐ and triacylglycerides (Figure 5).

### 3.5 Sphingolipids and Bile Acids Are Positively Associated With Lifespan‐Extension in Mice

In gonadal adipose tissues of female mice and in most tissues of males, many sphingolipids, specifically sphingomyelins and dihydroceramides, were found to be associated with higher levels in treatment groups with higher life‐extending effects (Figure 5). Interestingly, in kidney tissue of both male and female mice, ceramides and hexosylceramides also increased in response to lifespan‐extending interventions. Ceramide synthase enzymes catalyze the formation of (dihydro)ceramide and therefore affect the assembly and chain lengths of all sphingolipids. Specifically, longevity assurance (LASS) genes have been associated with ceramide‐depending longevity in model species. For selected cases, interaction of ceramides with transmembrane‐spanning domains of proteins have shown how sphingolipases may release sphingolipids into cell organelles as second messengers in signaling pathways (Zhang et al. 2023).

A further class of lipid metabolites, bile acids, was also found to be associated with increased lifespan (Figure 5). Bile acids are formed from cholesterol through transformation via both microbial proteins and liver enzymes. Groups of novel bile acids have recently been found in humans, possibly with signaling properties in distal organs (Quinn et al. 2020). Here, we found increasing levels of bile acids to be significantly associated with extended lifespan in male mice. In both liver and plasma, taurocholic acid and tauroursodeoxycholic acid were positively associated with longevity in male mice, and deoxycholic acid and campesterol sulfate (ST 28:1;O;S) in plasma of male mice. Bile acids are involved in lipid and carbohydrate metabolic pathway regulation, as well as insulin sensitivity (Arab et al. 2017). These positive benefits of bile acids suggest possible molecular interpretation of sexual dimorphisms, since positive associations are seen in male mice, but not female.

### 3.6 Other Compound Classes Show Less Dramatic Differences After Lifespan Interventions in Mice

Overall, carbohydrates showed negative associations across life‐extending treatments in plasma and liver (Figure 5). This finding is consistent with other studies, which have shown that caloric restriction causes liver glucose levels to drop (Kirk et al. 2009), while the liver prevents blood sugar levels from dropping too steeply through gluconeogenesis. Other carbohydrates also showed declines proportional to extended lifespan in male liver, including as maltose, sorbitol, dexoypentitol, lactose, sophorose, and fructose. In contrast, fructose‐6‐phosphate, fructose‐1‐phosphate, galactonic acid, phosphogluconic acid, myo‐inositol, trehalose, ribitol, galacto‐N‐biose, threonic acid, and glycerol‐N‐acetyl glucose were positively associated with increased lifespan in mice (Appendix S2). Hence, rather than responding as a class of compounds, these sugars responded in pathway‐specific manner to lifespan‐extending treatments. For example, the positive association of sugar phosphates indicated activation of glycolysis and pentose‐phosphate pathways, whereas negatively associated sugars comprised degradation products of foods, glycans and starch in addition to sugar alcohols that have been associated with cancers of the liver, kidney, and brain (Ismail et al. 2020). Hence, both trends may be interpreted as better use of food‐derived sugars, with less production of potentially harmful reduction products of sugar alcohols.

**FIGURE 7:**
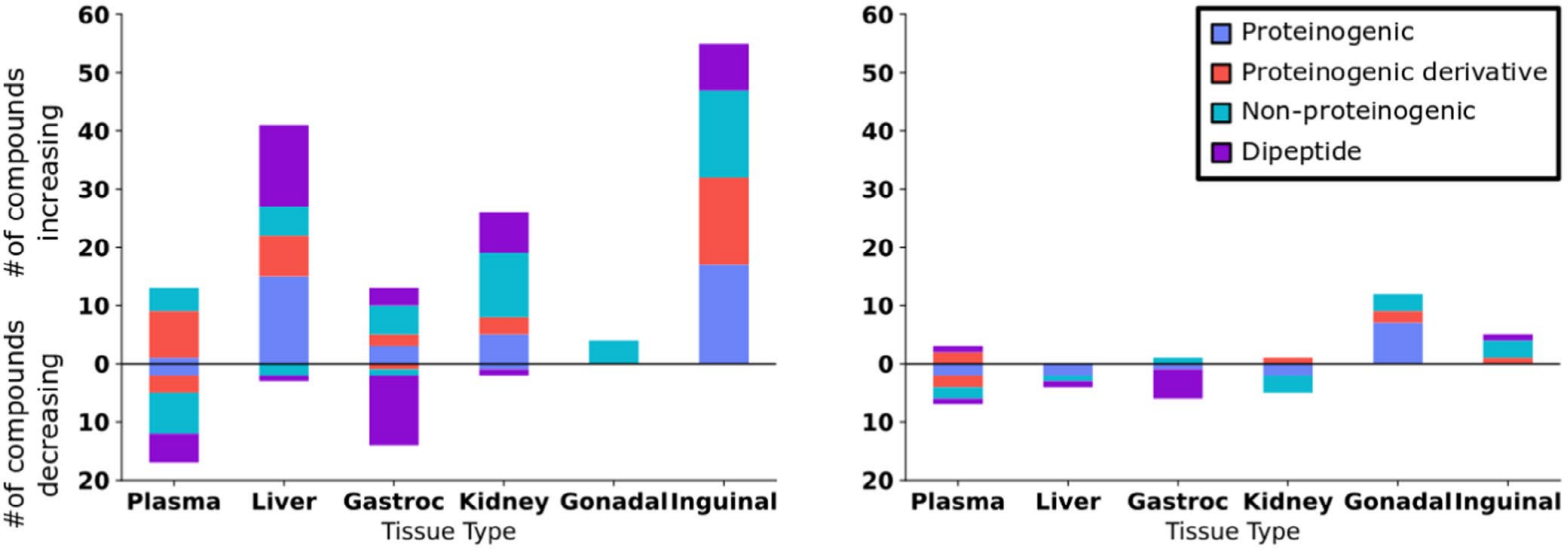
Subclassification of changes of amino acids and their derivatives across lifespan‐extending interventions. Average lifespan extensions of interventions were used for Jonckheere's trend test statistics at FDR *q* < 0.1. Left panel: Male mice. Right panel: Female mice. Amino acids were differentiated as dipeptides (purple), proteinogenic amino acids (red), non‐proteinogenic amino acids (dark blue) and proteinogenic amino acid derivatives (e.g., methylated, acetylated, hydroxylated, and sulfoxide variants).

Consistent with other studies, amino acids and dipeptides showed varying positive and negative associations with treatment‐induced lifespan extension, for both male and female mice and across various tissues (Figures 4 and 7, Appendix S1; Xie et al. 2022). Like lipids, amino acids and dipeptides serve various physiological functions. Beyond use for protein biosynthesis and as nutrients, amino acids have been shown to be involved in metabolic signaling (Canfield and Bradshaw 2019). For comparison, we grouped the 20 proteinogenic amino acids as separately from their modified derivatives, non‐proteinogenic amino acids, and dipeptides (Figure 7). These graphs demonstrate that amino acids were more affected by lifespan‐extending treatments in male than in female mice. When considering only proteinogenic amino acids in the different organs, we noted that lifespan‐extending effects were not simply explainable by differences in overall protein degradation, for example, by differences in metabolism of gastrocnemius muscle proteins (Figure 7). Although muscle dipeptides (which can be regarded as an indication of protein turnover) were diminished in proportion to lifespan‐extending treatments in both male and female mice, we found positive associations of dipeptides in male liver, inguinal fat, and kidney (Figure 7). Similarly, in these tissues, proteinogenic amino acids also responded with positive Jonckheere's trend test associations to lifespan‐extending treatments. Hence, it appears that muscle protein turnover was altered in the opposite direction to changes seen in liver and inguinal fat depots. In male mice specifically, the overall positive trend for amino acids and their derivatives was unexpected. While the total amount of protein content in the adipose tissues is modest in comparison with muscles, the relative changes in inguinal fat still show a sustained effect across all types of amino acid derivatives (Figure 7, left). In male inguinal fat, the number and diversity of amino acid derivatives with positive association to lifespan‐extending treatments was larger than for both dipeptides and classic proteinogenic amino acids. Similarly, plasma showed large changes in derivatives of amino acids, surpassing those of any other subclass. Hence, these data support the notion that amino acid derivatives (such as methylated and acetylated versions) may be under specific biological regulation and are not likely to be solely explained by changes in protein turnover. Other non‐proteinogenic amino acids (such as N‐omega‐acetylhistamine) also showed specific and separate effects not seen in either proteinogenic‐amino acids or their corresponding derivatives, for example, in male kidney and in male plasma (Figure 7). The exact function of those amino acids is as varied as their structure and warrants more detailed and function‐directed investigations.

## 4 Discussion

Previous studies investigating the drug effects on average lifespan increases (Table 1) were performed at three different locations using UM‐HET3 mice, while the life‐extending effects of caloric restriction reflect data from the Jackson Laboratory. The lifespan increases identified in the original studies were based on comparisons to same‐year control mice, whose lifespans vary from site to site and from year to year, and between males and females. Additionally, lifespan extension effects of 17‐α‐estradiol (17aE2) and acarbose (Aca) have only been investigated in UM‐Het3 mice. For these reasons, our estimated percent lifespan extension for each intervention group should be interpreted with some caution. Instead of confirming the extent of lifespan extensions, we focused on the hypothesis that the metabolic effects of lifespan‐extending treatments are not directly related to generic trends of metabolic changes during normal aging.

Across organs, we used both set enrichment statistics and Jonckheere's trend test to find general metabolite classes and specific compounds that responded most strongly to lifespan‐extending treatments. Consequently, we found lipids to be the most dramatically regulated class of metabolites across organs (Gonzalez‐Covarrubias 2013). Interestingly, while the relative percentage of significantly changed lipids was similar between female and male mice, our trend‐test analyses showed that most of these changes were associated with increases in lifespan only in male, but not in female mice. These similarities of metabolic trends across organs suggest that our data provide valuable physiological insights. A surprising fact, however, was the low number of metabolites to be differentially expressed between rapamycin and the age‐matched controls. While rapamycin has been repeatedly reported as an effective way to increase lifespan in mice (Siegmund et al. 2017), our data are supported by an earlier report that also found few significantly differentially expressed metabolites for rapamycin treated mice (Fok et al. 2014). This finding suggests that regulation of mTOR pathways may not always lead to dramatic changes of metabolite abundances. Instead, effects should be discussed with respect to differences found within each organ.

### 4.1 Adipose Tissues

Apart from liver and plasma, the organs with the most dramatic changes were the adipose tissues. Historically, adipose tissue has been considered primarily for energy storage. However, recent studies have shown different adipose tissue deposits carry out varying metabolic processes, and are a plausible target to better understand aging (Miller et al. 2017), including aging sexual dimorphism. Here, we found different responses between gonadal and inguinal fat tissues, and different magnitude and directions of lifespan‐associated changes in female and male mice. The lifespan‐extending treatments we used here decreased the glycerophospholipids levels in male gonadal fat, but strongly increased such lipids in female gonadal fat. While 17aE2 has been shown previously to be involved in regulating glycerophospholipid metabolism in a male mouse cell line, more work is needed to investigate these sexual dimorphisms of treatment effects (Liu et al. 2015). In the female mice, gonadal fat showed the most numerous positive associations, including increased proportions of glycerophospholipids, sphingolipids, and ether lipids with increased life extension effect of interventions. As caloric restriction has been shown to slow ovarian aging in mice, the effects we found in this study may be indicative of our interventions inducing a healthier gonadal fat pad, which may be connected to healthier ovarian aging (Nelson et al. 1995; Selesniemi et al. 2011; Finch 2014).

Certain classes of inguinal fat are important for maintaining insulin sensitivity, and our data suggest these life‐extending interventions induce increased levels of these classes (Tran et al. 2008; Cox‐York et al. 2015; Chang et al. 2023). Adipose tissues are classified as brown, beige, white, and pink (Richard et al. 2000) and have different functions, with brown adipose tissues being more sensitive to insulin (Esteve Ràfols 2014). While inguinal fat is generally considered a white adipose tissue, studies show calorie restriction (Li et al. 2023) leads to browning of white adipose tissues (Fabbiano et al. 2016; Sheng et al. 2021; Saely, Geiger, and Drexel 2012; Richard et al. 2000). As glycerophospholipids are more prevalent in brown adipose tissue than white adipose tissue (Leiria and Tseng 2020), the increasing levels of phospholipids in our results may support this notion of browning of inguinal fat in male mice under lifespan‐extending treatments.

Surprisingly, while triacylglycerides showed sexual dimorphic trends between male and female mice in inguinal and gonadal fat tissues, specifically for PUFA‐containing fats, this difference between the sexes was less for diacylglycerides. Hence, these data support the notion that PUFA‐specific acyltransferases were differentially modulated by the lifespan‐extending treatments in male and female mice, while generic lipase activities were associated with lifespan in a similar way for both sexes. PUFA‐containing diets have shown to improve healthy aging (Janssen and Kiliaan 2014; Cutuli 2017), and total PUFA levels have been associated with maintaining physical performance in aging subjects (Abbatecola et al. 2009). Our study adds to this observation, but suggests that lifespan‐extending interventions can make PUFAs more available, even under otherwise identical diets.

For female gonadal fat, and male inguinal fat, we found isoleucine and leucine levels to be increased in groups exposed to interventions with greater lifespan extension effects. Studies have shown supplemental leucine increases lifespan in yeast and *Caenorhabditis elegans* (Alvers et al. 2009). Additionally, leucine combined with vitamin D was shown to increase muscle strength in the elderly (Guo et al. 2022). Interestingly, leucine has been shown to activate the mTOR pathway (Edwards et al. 2015), raising the possibility of isoleucine and leucine as signaling molecules related to aging. Dipeptides have also been shown to be higher in brown adipose tissue, which is consistent with a slight increase in dipeptides in most treatments in female inguinal fat (Heidenreich et al. 2021), supporting our theory that adipose tissue browning increased across these life‐extending treatments.

### 4.2 Plasma and Liver

Overall, for plasma and liver, we found large effects of lifespan‐extending treatments on lipid metabolism that were generally similar for both male and female mice. The decrease of glycolipid levels, specifically di‐ and triacylglycerides, may have been caused by several factors. Variations in lipase activity and regulation can result in decreased fatty acid biosynthesis. This interpretation is supported by our finding of reduced levels of free fatty acids in the liver. Free fatty acids induce production of triacylglycerides in the liver (Niklas et al. 2012), which can contribute to fatty liver disease (Liu et al. 2016). While reduced caloric intake may explain reduced fatty acid levels in the CR group, altered lipase activity may play a role in the other interventions. Another possible explanation is altered expression of the de novo lipogenic pathway, which is the highly conserved pathway involved in converting sugars to fatty acids, primarily in liver and adipose tissues (Ameer et al. 2014). The de novo lipogenesis pathway has been shown to be regulated by the mTOR pathway (Laplante and Sabatini 2009), which is effected by these drug interventions (Jiang et al. 2023), making the combined altered effect of these two pathways a potential mechanism. While plasma lipid concentrations are previously shown to increase with age (Hornburg et al. 2023), we find here that lifespan‐extending treatments are associated with decreased amounts of these neutral lipids. High levels of plasma glycerolipids are a hallmark of conditions like hyperlipidemia and metabolic syndrome (Esteve, Ricart, and Fernández‐Real 2005), inducible by high fat‐ (Miao et al. 2015) or high‐carbohydrate diets (Wang, Yu, and Walzem 2008). Hence, the overall decrease of neutral (energy storage) lipids by lifespan‐extending treatments can be regarded as contributing to overall health.

Phospholipids in plasma were negatively associated with lifespan‐extending treatments, but the opposite trend was found for the liver. The decrease in such membrane lipids in plasma may be interpreted as a lower number of very‐low density lipoprotein particles released by the liver, as a consequence of lower liver lipogenesis. This conclusion is supported by the decrease of triacylglycerides in plasma. In both sexes, we noted decreased levels of LPCs in the plasma and liver, which is also a sign of liver health, because high levels of LPCs reduce mitochondrial function (Hollie et al. 2014). Because increased plasma phospholipids are associated with many diseases, including cardiovascular disease, diabetes, ovarian cancer, and renal failure, we interpret our data on phospholipids as evidence of better metabolic health in mice exposed to these anti‐aging interventions (Law et al. 2019). Consistent with our findings, previous studies have shown increased levels of phosphatidylethanolamine membrane lipids, along with decreased levels of fat and carbohydrates in the livers of calorically restricted male mice (Jové et al. 2014). In liver and plasma, a few associations were found for cholesterol esters and sphingolipids that showed some positive associations with increased lifespan, especially in male liver. We did not expect to see sphingomyelins increased in the male plasma treatments, as plasma sphingomyelins are associated with increased risk of coronary artery disease (Jiang et al. 2000). Our data do not show strong support for radical‐based oxidation damage to liver and plasma lipids. In plasma, no effects were noted on oxidized (radical‐damaged) versions of any lipid class, with some modest effects on oxidized versions of free fatty acids and triacylglycerides in the liver. Oxidation‐based damage has been long debated to be involved in aging and aging‐related diseases (Muller et al. 2007; Castello et al. 2010; Møller et al. 2010; Liguori et al. 2018; Elmansi and Miller 2023). Others argue against this theory, citing the lack of effect of administration of oxidized compounds and antioxidants on lifespan (Holloszy 1998; Lee et al. 2004; Gladyshev 2014; Sadowska‐Bartosz and Bartosz 2014). For regular beta‐oxidation of lipids, acylcarnitines can be viewed as surrogate for mitochondrial health (McCann et al. 2021). Overall, we observed modest decreases of acylcarnitines in plasma, along with modest increases of this lipid class in other organs. Hence, lifespan‐extending treatments showed limited, but positive, effects on mitochondrial beta‐oxidation.

### 4.3 Muscle

Overall, the effects of lifespan‐extending treatments on gastrocnemius muscle were far more limited than expected, and certainly less dramatic than effects found in adipose tissues, liver, or plasma. Modest increases in glycerophospholipids and ether membrane lipids were found in male mice, but not in females. While proteinogenic amino acids showed few differences under lifespan‐extending treatments, the levels of dipeptides were negatively associated with lifespan, consistent with a notion of reduced muscle protein content (Margolis et al. 2016). Additionally, muscle wasting has been shown to be associated with altered glycerophospholipid profiles (van der Veen et al. 2017; Senoo et al. 2020). In combination, our muscle dipeptide and membrane lipid data support this interpretation.

### 4.4 Kidney

In the kidney, many metabolite classes, including carbohydrates, free fatty acids, membrane lipids, and PUFA‐containing triacylglyerides, are positively associated with increased lifespan, with more associations in male than female mice. Previous studies have shown correlations between kidney lipid levels and aging (Tsugawa et al. 2024). Generally, kidney function decreases with age (Davies and Shock 1950; Jiang et al. 2012; Jiao et al. 2022). Jiao et al. showed aging affects amino acid metabolism, carbohydrate metabolism, and some glycerolipid and glycerophospholipid metabolism in mouse kidneys (Jiao et al. 2022). However, anti‐aging studies have shown interventions that increase longevity also increase kidney health (Bolignano et al. 2014). These interventions include known players in aging, including sirtuins (SIRTs) and the mTOR pathway (Ma, Yung, and Chan 2018; Yacoub, Lee, and He 2014; Hasegawa et al. 2010; Huber, Walz, and Kuehn 2011). The mTOR pathway is being considered as a target pathway to treat kidney disease (Gui and Dai 2020). Our data also show an increase in sphingolipids, which have been shown to be important in maintaining kidney health (Mallela, Merscher, and Fornoni 2022). High ceramide levels in plasma are correlated with kidney disease (Weinberg 2006). Interestingly, when we analyzed data only based on treatment effects (without Jonckheere's trend test), ceramide and sphingolipid levels decreased across treatments in the male mice. The lowest levels were found both in the 4‐month‐old controls and in the caloric restriction treatment. This suggests that the drug treatments do not work as effectively as caloric restriction in reducing sphingolipid levels in the kidneys. As there were minimal significant effects on sphingolipids in plasma, these data may suggest that damage accumulates in the kidney well before the metabolic changes are detected in blood. Similarly, an inverse relationship between plasma and kidney ceramide levels was reported before, supporting our data (Sas et al. 2015).

## 5 Conclusion

Understanding the metabolomics of healthy aging, especially when it leads to exceptional longevity, provides a wealth of knowledge for overall health for everyone. This study highlighted lipids as strongly affected by interventions shown to induce life extension. For more detailed investigations, lipidomics data will need to be interpreted with respect to both biochemical and physiological effects. Lipids are not only an essential part of nutrition, used for cell homeostasis from energy to membrane stability, but they often act as signals to regulate cellular processes. Tools currently focus on lipidomic pathways, but signaling and lipid actions as regulators are mostly missing from databases, or only give general guidance without specific molecular targets or organ specificity. Our study also re‐emphasizes the notion that functions of adipose tissues extend well beyond energy storage, suggesting adipose deposits are not only metabolically active, but uniquely differ from one another. Our study is the first to highlight the distinct role of adipose deposits and their specific roles in healthy aging and longevity, including their sexual dimorphism.

## Author Contributions

S.G. involved in data acquisition and processing, data analysis, data interpretation, and manuscript writing. N.C.S. involved in data acquisition and processing. L.B. involved in data acquisition and processing. Z.R. involved in data acquisition and processing. D.C.S. involved in data analysis. A.O.A. involved in data analysis. R.A.M. involved in study design, mouse husbandry, data interpretation, and manuscript writing. O.F. involved in study design, data interpretation, and manuscript writing.

## Conflicts of Interest

The authors declare no conflicts of interest.

## Supporting information


Appendix S1

Appendix S2

Appendix S3

## Data Availability

Data are available via the ELITE Portal (https://eliteportal.synapse.org). The ELITE Portal is a platform for accessing data, analyses, and tools generated by the Exceptional Longevity projects funded by the National Institute on Aging (NIA) to enable open‐science practices and accelerate translational learning. The data, analyses, and tools are shared early in the research cycle without a publication embargo on secondary use. Data are available for general research use according to the following requirements for data access and data attribution (https://eliteportal.synapse.org/DataAccess). For access to content described in this manuscript, see: https://doi.org/10.7303/syn64322332.
