# Supplementary material for: Drug‐Based Lifespan Extension in Mice Strongly Affects Lipids Across Six Organs
Source: Aging Cell. 2025 Mar 24;24(5):e14465. doi: 10.1111/acel.14465 (PMC12073903; doi:10.1111/acel.14465)
Supplement: Supplementary file 1 — Appendix S1 [file ACEL-24-e14465-s002.pdf]

**Supplement 1: Drug-based lifespan extension in mice strongly affects lipids across six organs.**

Sara Greenfield[1]; Nathaniel C Stevens[1]; Lauren Bishop[1]; Zachary Rabow[1]; Daniela C. Soto[3], Abdali Omar Abdullah[1]; Richard A. Miller [2]; Oliver Fiehn[1]

[1] West Coast Metabolomics Center, University of California, Davis, CA 95618, USA

[2] Department of Pathology and Geriatrics Center, University of Michigan, Ann Arbor, MI 48103, USA

[3]Department of Biochemistry & Molecular Medicine, MIND Institute, University of California, Davis, CA 95616, USA

Supplemental Methods

**Sample preparation:** Except for plasma and adipose tissues, samples were lyophilized using Labconco FreeZone lyophilizer (Kansas City, MO) and homogenized using a Geno/Grinder 2010 Spex (Cole-Parmer, Metuchen, NJ ) prior to extraction. For the tissues,1 mg dry weight (DW) kidney, 2 mg DW liver, 3 mg DW gastrocnemius muscle, and 5 mg DW gonadal and inguinal fat were measured by a Mettler-Toledo (Columbus, OH) XP105 DeltaRange ultrafine balance to 0.1mg accuracy. Powdered lyophilized tissues were extracted using 1 mL of a -20°C cold ternary solvent method of methanol, water and MTBE for liquid extraction (Matyash 2008) to yield a lipophilic phase and a hydrophilic bilayer phase. 20 µL of plasma were thawed on ice and extracted with the same method as the tissues. Mouse plasma bioreclamation controls and method blanks were extracted alongside the samples. A mixture of 76 internal standards (UltimateSPLASH ONE kit, plus acylcarnitines and free fatty acids; Supplemental Table 2) from Avanti Polar Lipids (Alabaster, AL) and Cambridge Isotope Laboratories (Tewksbury, MA) prepared in 225 µL methanol was added to each sample, vortexed for 20 s, followed by addition of 750 µL MTBE. Two stainless steel beads were added to each sample, and shaken for 5 minutes. Phase separation was achieved by adding 188 µL of LCMS-grade water, vortexed for 20 s, shaken for 6 min, and centrifuged at 14,000 x *g* for 2 minutes. 2 aliquots of 350 µL of the lipophilic (top) layer (175 µL for adipose tissues) were used for lipidomic analysis. The hydrophilic (bottom) layer was separated into 2 aliquots of 110 µL and dried for hydrophilic compound analysis using HILIC-MS/MS and GC-TOF MS. The remaining fractions of each sample were combined to create quality control pools (QC) that were injected after each set of 10 samples and used for data normalization. All extracts were desiccated to total dryness and stored at -20°C before data acquisition by mass spectrometry.

**Data acquisition:** (a) Primary metabolism by Gas chromatography - time of flight mass spectrometry (GC-TOF MS). Samples were derivatized to increase thermostability and to prevent mutarotation for sugars. Derivatization used methoxyamination using 10 µL of a methoxyamine pyridine solution (40 mg/mL), shaken at 30°C for 90 minutes. 10 µL of a solution of internal standard markers (C08-C30 fatty acid methyl esters) was added to 1 mL of N-methyl-N-trimethylsilyltrifluoroacetamide (MSTFA). 90 µL of this MSTFA solution was added to the samples and shaken at 37°C for 30 minutes. Samples were transferred to glass vials with micro-inserts. GC-TOF MS data acquisition was performed by injecting 0.5 µL with 25 s splitless time on an Agilent 7890 gas chromatograph using a Restek Rtx-5Sil MS column with an Intra-Guard (30m x 0.25 mm i.d. x 0.25 um df) and 1mL/min Helium flow. The temperature gradient started at isocratic 50°C for 30s, increased by 20°C/min to 330°C, final hold time, 10 min. Mass spectrometry was performed using a Leco Pegasus IV mass spectrometer at 70 eV at 17 Hz from 80-500 Da at 1850 V detector voltage. Blank samples, pool QC samples and BioIVT mouse plasma QC samples were injected after every set of 10 samples. Data processing was performed by peak detection, baseline subtraction and spectral deconvolution using Leco ChromaTOF vs 4.50 followed by Binbase metabolite annotation and reporting.

(b) Biogenic amines by hydrophilic interaction chromatography-accurate mass tandem mass spectrometry (HILIC-MS/MS). Samples were resuspended in 100 µL of 80:20 ACN:H2O with CUDA and 25 internal standards (TABLE 1). Samples were resuspended in the starting LC buffer (mobile phase B, below). 5 µL were injected into a Waters BEH Amide 1.7um, 2.1x150mm column with a BEH Amide (5 mm × 1.2 mm, 1.7 µm particle size) pre-column (Waters, Milford MA). Mobile

phase A consisted of 100% H2O +10 mM ammonium formate + 0.125% formic acid, and mobile phase B consisted of 95:5 ACN/H2O + 10 mM ammonium formate + 0.125% formic acid. The LC mobile phase gradient started at 100% B for 0.0-2.0 minutes, decreased to 70% B from 2.0-7.7 minutes, decreased to 40% B from 7.7-9.5 minutes, increased to 30% B from 9.5-10.25 minutes, increased to 100% B from 10.25-12.75 minutes, and held at 100% B from 12.75-17.0 minutes. (c) Complex lipids by reversed phase chromatography-accurate mass tandem mass spectrometry (RPLC-MS/MS). Samples were resuspended in 100 µL of 90:10 MeOH:TOL with CUDA. Lipidomic analysis was performed by RPLC-MS/MS using a ThermoFisher Vanquish Focus UHPLC with mass spectra collected with a QExactive HF+ or QExactive HFX from 120-1700 m/z in positive and negative electrospray with data dependent MS/MS acquisitions. A Waters Acquity UPLC CSH C18 1.7 um 2.1 x 100 mm column was used with a Waters Acquity CSH C18 1.7 um 2.1 x 5 mm precolumn with a 12-minute acetonitrile/water/isopropanol ternary gradient using formic acid and ammonium formate (positive ESI) or ammonium acetate (negative ESI) as pH buffers. For positive mode ionization, Mobile phase A consisted of acetonitrile/water (60/40, v/v) with 0.1% formic acid and 10 mM ammonium formate as modifiers, and Mobile phase B consisted of isopropanol/acetonitrile (90:10, v/v) with 0.1% formic acid and 10 mM ammonium formate. For negative mode ionization, Mobile phase A consisted of acetonitrile/water (60/40, v/v) with 10 mM ammonium acetate as modifier, and Mobile phase B consisted of isopropanol/acetonitrile (90:10, v/v) with 10 mM ammonium acetate as modifier. The LC mobile phase gradient started at 15% B, increased to 30% B from 0.0-2.0 minutes, increased to 48% B from 2.0-2.5 minutes, increased to 82% B from 2.5-11.0 minutes, increased to 99% B from 11.0-11.5 minutes, held at 99% B from 11.5-12.0 minutes, returned to 15% B from 12.0-12.1 minutes and held at 15% B from 12.1-14.2 minutes. The autosampler was held at 4 degrees C for plasma, gastroc, gonadal and inguinals fats, and 10 degrees C for liver and kidney. Needle wash was performed using isopropanol for 10 seconds after each sample injection. Spectra data was collected in a scan range of approximately 120-1700m/z. See supplemental table 3 for injection volumes.

**LC-MS/MS data processing.** Retention time drifts were corrected for by internal standards in the method blank. Peak detection and reporting, including gap filling, was performed by MS-DIAL v. 4.48 (Tsugawa 2015). Compound identification was performed by accurate mass and MS/MS spectral matching using MassBank of North America (MassBank.us) and NIST20 libraries (NIST, Gaithersberg, MD), in addition to retention time curation (Bonini 2020 ). Peaks were removed at s/n <5 or signal intensities <10,000. MS-FLO software was used to integrate adducts and remove duplicates (DeFelice 2017). Datasets were normalized by SERRF normalized against the pooled samples (Fan 2019). Raw data and curated data is available on the Metabolomics workbench.

**Supplemental Table 1:** Numbers of mice measured per treatment, tissue, and sex. Male = M; Female = F

| Treatment | Plasma | Muscle | Kidney | Liver | Gonadal | Inguinal |
|---|---|---|---|---|---|---|
| 12 month old Controls | 24M / 24F | 23M / 19F | 25M /24 F | 25M / 23F | 25M / 24F | 25M / 24F |
| 4 month old controls | 14M / 12F | 13M / 12F | 13M / 12F | 14M / 12F | 14M / 12F | 14M / 12F |
| Caloric Restriction (CR) | 14M / 11F | 13M / 7F | 14M / 11F | 14M / 11F | 14M / 9F | 14M / 9F |
| Acarbose (Aca) | 14M / 12F | 13M / 7F | 14M / 12F | 14M / 12F | 14M / 12F | 14M / 12F |
| Estradiol (17ae3) | 14M / 12F | 13M / 8F | 14M / 12F | 14M / 12F | 14M / 12F | 14M / 12F |
| Rapamycin (Rapa) | 13M / 11F | 11M / 8F | 11M / 9F | 13M / 12F | 13M / 12F | 13M / 12F |
| Canagliflozin (Cana) | 14M / 12F | 13M / 8F | 14M / 12F | 14M / 12F | 14M / 13F | 14M / 13F |

**Supplemental Table 2:** Hydrophilic Interaction Chromatography-accurate mass tandem mass spectrometry (HILIC-MS/MS) - internal standards

| Common Name | MS1 m/z | mzrt RT |
|---|---|---|
| 1_15N2-L-Arginine iSTD [M+H]+ | 177.113 | 9.454084296 |
| 1_CUDA iSTD [M+H]+ | 341.2799 | 1.22676137 |
| 1_D10-Isoleucine iSTD [M+H]+ | 142.164 | 7.212536585 |
| 1_D10-Leucine iSTD [M+H]+ | 142.164 | 7.03560374 |
| 1_D2-Ornithine iSTD [M+H]+ | 135.109 | 9.594360782 |
| 1_D3-1-Methylnicotinamide iSTD [M]+ | 140.0898 | 6.156326424 |
| 1_D3-ACar(2:0) iSTD [M+H]+ | 207.1419 | 7.2333184 |
| 1_D3-Aspartic Acid iSTD [M+H]+ | 137.0633 | 9.225601503 |
| 1_D3-Creatine iSTD [M+H]+ | 135.0956 | 8.075596711 |
| 1_D3-Creatinine iSTD [M+H]+ | 117.085 | 4.981616413 |
| 1_D3-DL-Alanine iSTD [M+H]+ | 93.0738 | 8.099020663 |
| 1_D3-DL-Glutamic acid iSTD [M+H]+ | 151.0793 | 8.75178728 |
| 1_D3-Histamine, N-methyl- iSTD [M+H]+ | 129.1214 | 7.343200654 |
| 1_D3-L-Carnitine iSTD [M+H]+ | 165.1313 | 7.71498059 |
| 1_D3-Serine iSTD [M+H]+ | 109.068 | 8.657321527 |
| 1_D4-Alanine iSTD [M+H]+ | 94.081 | 8.097328725 |
| 1_D4-Cystine iSTD [M+H]+ | 245.056 | 9.89673717 |
| 1_D5-Glutamic acid iSTD [M+H]+ | 153.0915 | 8.765266046 |
| 1_D5-Histidine iSTD [M+H]+ | 161.1079 | 9.382629183 |
| 1_D5-L-Glutamine iSTD [M+H]+ | 152.1078 | 8.569204307 |
| 1_D5-Threonine iSTD [M+H]+ | 125.097 | 8.264420551 |
| 1_D7-Arginine iSTD [M+H]+ | 182.1626 | 9.450108342 |
| 1_D7-Proline iSTD [M+H]+ | 123.114 | 7.822060815 |
| 1_D8-Lysine iSTD [M+H]+ | 155.163 | 9.542623043 |
| 1_D8-Methionine iSTD [M+H]+ | 158.108 | 7.418974343 |
| 1_D8-Phenylalanine iSTD [M+H]+ | 174.1362 | 6.888166565 |
| 1_D8-Tryptophan iSTD [M+H]+ | 213.147 | 6.839021511 |
| 1_D9-Betaine iSTD [M+H]+ | 127.1427 | 7.174820754 |
| 1_D9-Choline iSTD [M]+ | 113.1635 | 5.177851163 |
| 1_D9-TMAO iSTD [M+H]+ | 85.1322 | 5.5602688 |
| 1_Val-Tyr-Val iSTD [M+H]+ | 380.218 | 6.9227377 |
| 1_D8-Valine iSTD [M+H]+ | 126.1362 | 7.711928076 |
| 1_D7-Tyrosine iSTD [M+H]+ | 189.1250 | 7.653873001 |
| 1_D3-Asparagine iSTD [M+H]+ | 136.0790 | 8.695240409 |

Supplemental Table 3: Injection Volumes

| Biological Matrix | Plasma | Liver | Gastroc | Kidney | Gonadal | Inguinal |
|---|---|---|---|---|---|---|
| Tissue amount | 2 uL | 2 mg | 3 mg | 1 mg | 5 mg | 5 mg |
| Inj. Vol. - HILIC+ | 5 uL | 3 uL | 5 uL | 5 uL | 5 uL | 5 uL |
| Inj. Vol. - HILIC- | 5 uL | | | | | |
| Inj. Vol. - CSH+ | 2 uL | 3 uL | 2 uL | 2 uL | 1 uL | 1 uL |
| Inj. Vol. - CSH- | 5 uL | 5 uL | 3 uL | 2 uL | 3 uL | 3 uL |
| Inj. Vol. - GC | 0.5 uL | 0.5 uL | 0.5 uL | 0.5 uL | | |

Supplementary Figures

A.

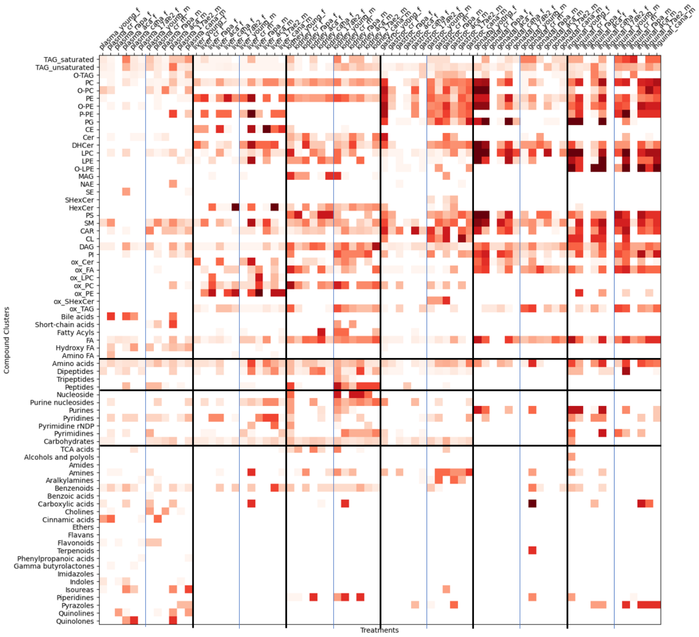


B.

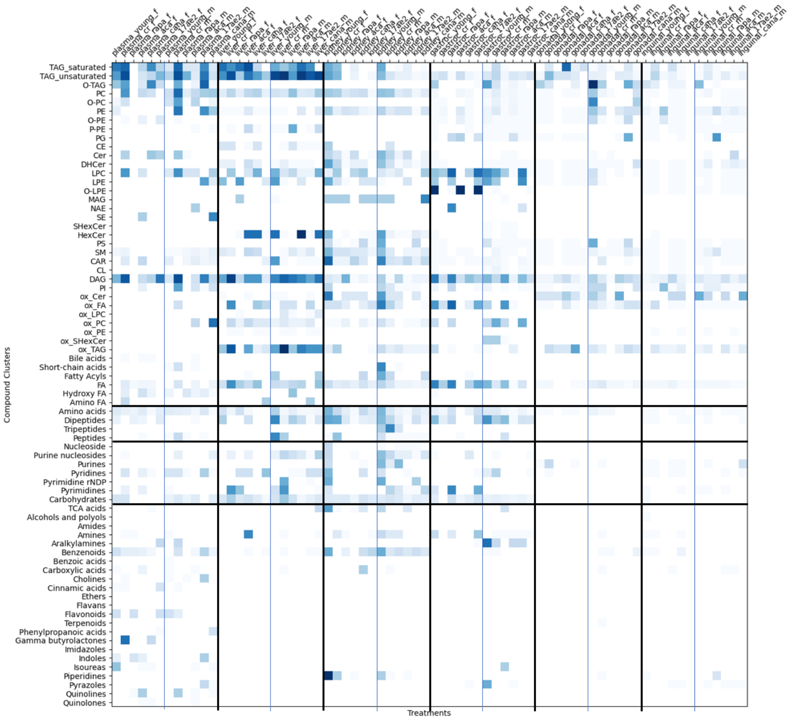


**Supplemental Figure 1. Effects of interventions on mouse organ metabolism by chemical set enrichment analyses**. Raw p-values from univariate statistical differences for interventions in 12-month-old mice were summarized into ChemRICH set statistics at Kolmogorov-Smirnov $p<0.05$. In Figure 3, to highlight classes with strong directional trends, we then subtracted the number of compounds significantly down regulated from the number significantly upregulated, and divided that number by the total number of compounds in that class for that tissue (Figure 3). To recognize compound clusters that are half and half

upregulated and downregulated, here we show a heatmap for the number of up-regulated compounds divided by the total number of compounds in that class for that tissue (A) and the number of down-regulated compounds in that cluster divided by the total number of compounds in that class for that tissue (B). Differential regulation is shown for primarily up-regulated classes (red) versus primarily down-regulated classes (blue). Color intensities indicate the percentage of differentially regulated compounds in the majority direction per class. Treatments are separated by sex and ordered by increasing life extension effect of treatment as listed in Table 1, starting with 4-month-old mice as a reference for biologically younger mice in each organ/sex column.
